# Lysine Residue at Position 22 of the AID Protein Regulates Its Class Switch Activity

**DOI:** 10.1371/journal.pone.0030667

**Published:** 2012-02-20

**Authors:** Roland Geisberger, Michael Huemer, Franz J. Gassner, Nadja Zaborsky, Alexander Egle, Richard Greil

**Affiliations:** Laboratory for Immunological and Molecular Cancer Research, IIIrd Medical Department with Hematology, Medical Oncology, Hemostaseology, Rheumatology and Infectiology, Paracelsus Medical University Salzburg, Salzburg, Austria; National Institute on Aging, United States of America

## Abstract

**Background:**

Activation induced deaminase (AID) mediates class switch recombination and somatic hypermutation of immunoglobulin (Ig) genes in germinal centre B cells. In order to regulate its specific activity and as a means to keep off-target mutations low, several mechanisms have evolved, including binding to specific cofactors, phosphorylation and destabilization of nuclear AID protein. Although ubiquitination at lysine residues of AID is recognized as an essential step in initiating degradation of nuclear AID, any functional relevance of lysine modifications has remained elusive.

**Methodology/Principal Findings:**

Here, we report functional implications of lysine modifications of the human AID protein by generating a panel of lysine to arginine mutants of AID and assessment of their catalytic class switch activity. We found that only mutation of Lys22 to Arg resulted in a significant reduction of class switching to IgG1 in transfected primary mouse B cells. This decrease in activity was neither reflected in reduced hypermutation of Ig genes in AID-mutant transfected DT40 B cell lines nor recapitulated in bacterial deamination assays, pointing to involvement of post-translational modification of Lys22 for AID activity in B cells.

**Conclusions/Significance:**

Our results imply that lysine modification may represent a novel level of AID regulation and that Lys22 is important for effective AID activity.

## Introduction

Activation induced deaminase (AID) induces class switch recombination (CSR) and somatic hypermutation (SHM) of Ig genes in B cells during the germinal centre reaction [1], [2], [3]. CSR changes the constant region of the antibody, thereby changing its effector function, whereas SHM increases the affinity of the antibody towards specific antigen by mutating the variable domain followed by selection of high affinity variants. AID initiates both processes by deaminating cytosines within the Ig genomic DNA, thereby generating uracils, which lead to DNA mismatches [4], [5], [6]. These mismatches are either processed into dsDNA breaks for CSR, or into mutations for SHM [7], [8].

Since AID is a DNA-mutating enzyme, its activity has to be tightly controlled to avoid collateral damage. Although AID is primarily targeted to the Ig locus for exerting its mutating function, leakiness of this process is evidenced by AID- mediated off-target mutations in non-Ig genes, including a panel of proto-oncogenes like *c-myc*, *Bcl6* and *p53* [9], [10]. In addition, as a result of mistargeted CSR, AID also contributes to the translocation of proto-oncogenes into the Ig locus, thereby accelerating malignant transformation and tumorigenesis [11], [12]. To ensure that AID-activity is largely restricted to the Ig locus, AID is regulated on different levels including (i) transcription [13], [14], [15], (ii) phosphorylation of residues T27, S38, Y184 and T140 [16], [17], [18], (iii) by binding of partner proteins to AID (reviewed in [8], (iv) by protein stability and (v) by subcellular localization [19], [20], [21].

Though AID acts on genomic DNA, it is largely restricted to the cytoplasm due to a strong C-terminal Exportin/Crm1-dependent nuclear export sequence (NES), which can be blocked by leptomycin B (LMB) [22], [23], [24]. Deletion or mutation of the NES leads to nuclear accumulation of AID, based on active import, which is mostly dependent on the structural integrity of AID and independent of a defined nuclear localization sequence (NLS) [21]. Interestingly, while deletion of the NES leads to nuclear accumulation and an increased mutational activity at Ig- as well as non-Ig genes, the CSR-activity of AID is strongly impaired [25]. Additionally, nuclear accumulation is accompanied by degradative ubiquitination and decreased protein stability [20], [19]. Based on these findings, it is supposed that AID is restricted to the cytoplasm to minimize the level of off-target mutations and to allow the production of stable AID protein, which is necessary for CSR.

Although it could be previously shown that lysine residues within the AID protein are ubiquitinated when nuclear export is blocked, functional implications of lysine modifications have not been assessed [19]. In our study, we investigated whether modification of lysine residues within the AID protein is important for CSR and SHM activity. Using a lysine to arginine mutagenesis screen, we found that mutating Lys22 to Arg (termed mutant K3) hampers CSR activity in B cells. However, despite of reduced activity in CSR, mutant K3 was active to wildtype levels in somatic hypermutaion and in bacterial deamination assays. Our experiments suggest a role of Lys22 in potentiating CSR activity of AID, providing evidence for a novel level of AID regulation.

## Results

### CSR activity of AID lysine to arginine mutants

**Figure 1:**
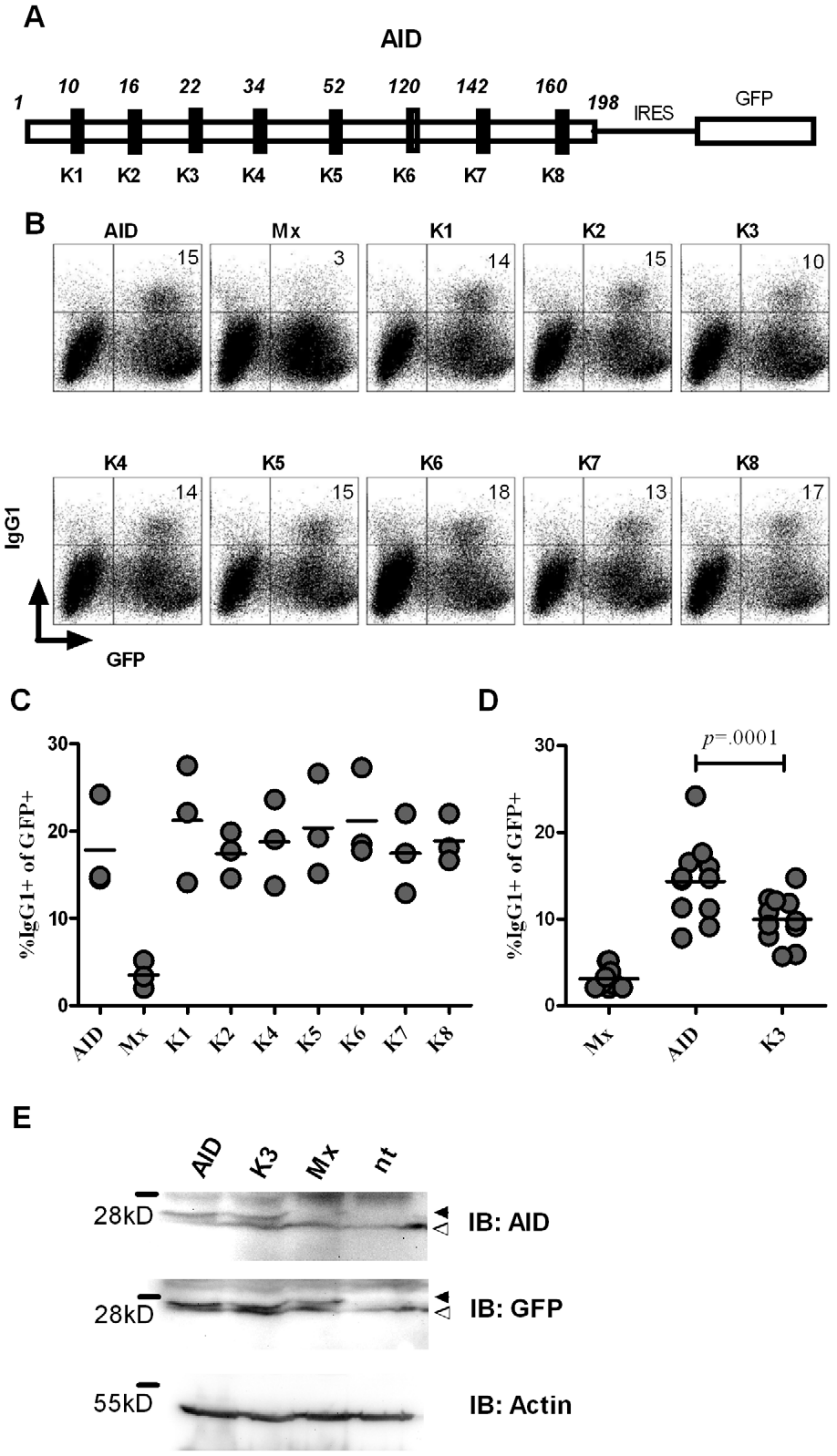
Effect of lysine to arginine mutations in the AID protein on CSR activity. *(A)* Schematic representation of AID highlighting the position of the lysine (K) residues. For CSR assays, untagged constructs were expressed bicistronically together with GFP separated by an internal ribosome entry site (IRES). *(B)* Representative FACS profile for CSR activity of individual Lys to Arg mutants. B cells from AID deficient mice were retrovirally transfected with the respective lysine AID mutant (Mx: empty vector) and CSR to IgG1 was determined upon stimulation of cells with LPS and IL-4. GFP expression of infected cells was driven from an IRES element located 3′ of the stop codon of the cloned AID mutant. The percentage in the upper right quadrants indicate the percentage of IgG1^+^ cells among the GFP^+^ (i.e. retrovirally infected) population. *(C)* Results from three independent experiments of a screen of lysine mutants showing the percentage of IgG1^+^ cells among the GFP^+^ population. *(D)* Results from 11 independent experiments comparing the CSR activity of AID and K3. *(E)* Western Blot on lysates from B cells retrovirally transfected with the respective constructs. Black arrowheads indicate specific band. White arrowheads indicate nonspecific band. (IB: immune blot).

As depicted in figure 1A, the human AID protein comprises eight lysine residues. To dissect the involvement of lysine modification in AID activity, we performed a mutational analysis by generating individual lysine to arginine mutants (K1 to K8, Fig. 1A) and tested the resulting mutants in class switch assays. Therefore, we retrovirally transfected primary AID deficient B cells with the respective AID mutants (K1 to K8) and determined class switching to IgG1 upon stimulation with LPS and IL-4. Interestingly, only mutation of lysine at position 22 (mutant K3) showed an apparent effect on CSR activity (Fig. 1B, C and D), which prompted us to more thoroughly investigate the influence of this residue on CSR in higher sample numbers. We could show that mutating Lys22 significantly affected CSR activity, leading to a decrease from 14.3% for AID, to 10.0% for K3 of IgG1 switched B cells (two-tailed paired t-test, p = 0.0001, n = 11, Fig. 1B and D).

### Enzymatic activity of mutant K3 is not perturbed

**Figure 2:**
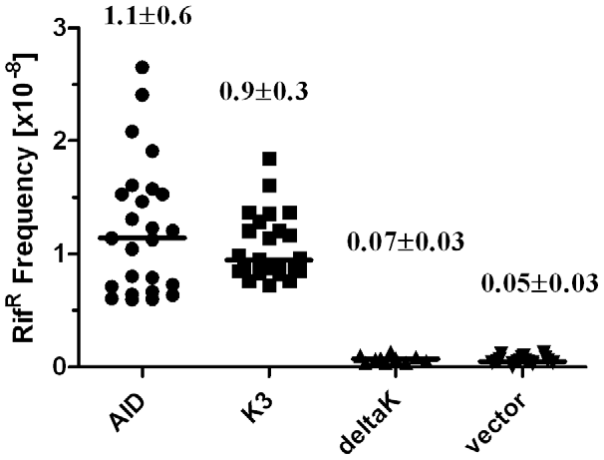
Mutational activity of Lys22 mutants. Rifampicin (Rif) resistance assay for AID-mediated cytidine deamination in UNG-deficient BW310 *E.coli*. The graph shows the mutation frequency (defined as the frequency of Rif-resistant clones) of individual starting colonies from *E.coli* transfected with the empty vector or plasmids expressing wt AID, K3 or lysineless deltaK. Median values ± SD are indicated in the graph.

As mutant K3 showed a decrease in CSR activity, we next asked whether this decrease is reflected in impaired biochemical activity of the mutant per se, irrespective of a putative lysine modification at this position. We therefore tested mutant K3 and wildtype AID for cytidine deaminase activity in *ung* deficient *E.coli* cells, determined by reversion of an inactive Rif resistance gene [4]. We found a similar frequency of Rif^r^ colonies in *E.coli* cells expressing K3 mutant as opposed to wildtype AID, showing that mutant K3 is catalytically active at a similar level as wildtype AID (Fig. 2). However, lysineless AID mutant in which all eight lysine residues are mutated to arginine (mutant deltaK) has lost enzymatic activity, probably due to structural destabilization (Fig. 2).

### Mutating Lys22 does not impair nuclear import

**Figure 3:**
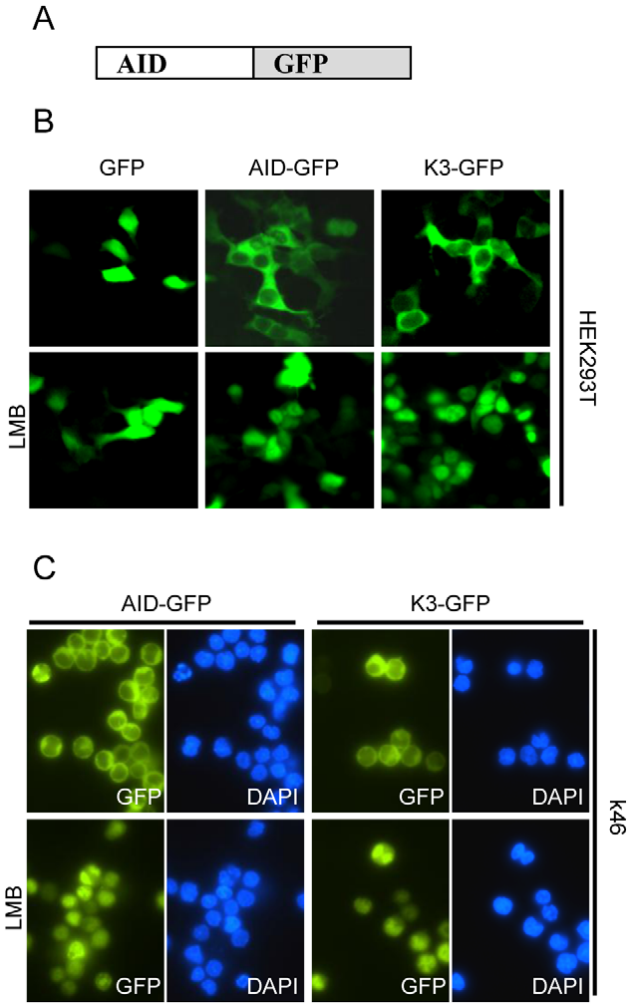
Subcellular localization of K3 is not different from wildtype AID. *(A)* Schematic representation of AID-GFP fusion constructs used for localization assays. GFP is fused to the C-terminus of AID or mutant K3. *(B)* HEK293T cells were transiently transfected with the respective GFP-fusion constructs. Two days post transfection, cells were treated as indicated with Leptomycin B (LMB) for 3 hours. Cells were fixed and localization of the respective GFP fusion proteins visualized using fluorescence microscopy. *(C)* The k46 B cell line stably expressing AID-GFP or K3-GFP fusion proteins were treated with or without Leptomycin B (LMB) for 3 hours and localization of GFP fusion constructs was determined using fluorescence microscopy.

Because Lys22 is located within a region of the AID protein previously found to be responsible for nuclear import [21], [23], we assessed whether mutation of Lys22 to Arg would interfere with nuclear/cytoplasmic shuttling of AID, which we speculated to be causative for the observed decrease in CSR activity. We found that in steady-state conditions, GFP-fusions of AID and mutant K3 were strictly localized to the cytoplasm of transfected B (mouse B cell line k46) and non-B cells (HEK293T) (Fig. 3). Nuclear accumulation of GFP-tagged AID and K3 could be induced by inhibition of nuclear export using LMB, indicating that nuclear import of AID is not impaired by Lys22Arg mutations (Fig. 3).

### Mutant K3 is active in hypermutation

**Figure 4:**
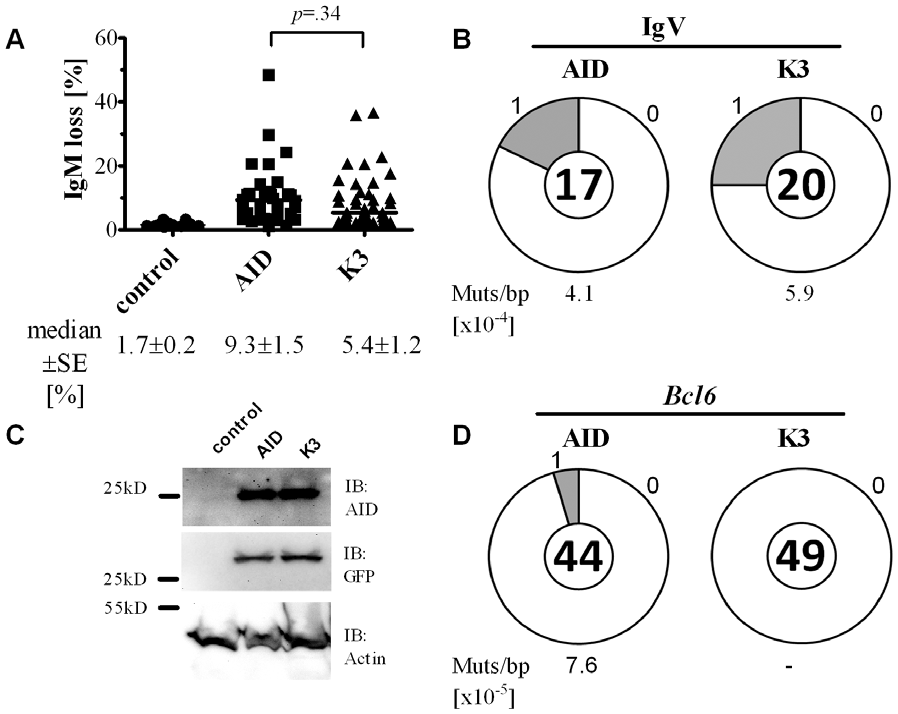
Somatic Hypermutation of IgV genes by wt AID and mutant K3. *(A)* Somatic hypermutation reflected as loss of IgM expression in AID^−/−^ ΨV^−/−^ IgM^+^ DT40 cells. AID^−/−^ ΨV^−/−^ IgM^+^ DT40 cells were stably transfected with plasmids encoding AID or K3 coupled to IRES-GFP expression. The percentage of sIgM-loss variants of GFP^+^ cells is expressed as the median ± SE of multiple (n) independent clonal transfectants determined three weeks after transfection (untransfected control, n = 12; AID, n = 36; K3, n = 44). *(B)* Proportion of sequences carrying mutations in IgV genes of DT40 cells. IgV regions were cloned from cDNA of AID and K3 expressing DT40 transfectants. Segment sizes in the pie charts are proportional to the number of sequences carrying the number of mutations indicated in the periphery of each chart. The frequency of mutations per bp sequenced and the total number of independent sequences analyzed is indicated underneath and in the center of each chart, respectively. *(C)* The abundance of AID and K3 in DT40 transfectants was determined by Western blotting from cell extracts. Blots were reprobed for GFP expression and actin levels as loading control. *(D)* Proportion of sequences carrying *Bcl6* mutations in AID deficient B cells retrovirally transfected with AID or K3 constructs, respectively. *Bcl6* was cloned from genomic DNA of sorted mouse B cells, retrovirally transfected to express AID or K3, respectively. Pie charts were generated as described in (B).

To determine the mutational activity of K3 in eukaryotic cells, we tested its ability to hypermutate an immunoglobulin V region gene in the B cell line DT40. Therefore, we transfected AID^−/−^ ΨV^−/−^ IgM^+^ DT40 cells with AID and K3 constructs, respectively and determined V region diversification by measuring the number of IgM-loss variants in several independent clones. We found similar activity in SHM from K3 transfected cells compared to those expressing wildtype AID (10.6% vs 8.7% mean IgM-loss; 9.3% vs 5.4% median IgM-loss; p = 0.34, unpaired two-tailed t-test; Fig. 4A). Also, protein levels were similar for wildtype and mutant K3 (Fig. 4C). To more thoroughly determine the mutational activity of K3, we sorted for AID or K3 expressing DT40 variants and analyzed the mutations within the V-region. We found that the amount of mutations within the V-region was similar for AID (3 out of 21 sequences mutated) and K3 (5 out of 24 sequences mutated) expressing DT40 cells, with no apparent difference in hotspot preference being observed (Mutation frequency 4.1 vs 5.9×10^−4^; p = 0.58, unpaired two-tailed t-test; Fig. 4B and S1). To test off-target activity of AID versus K3, we analysed mutations within the intronic region of *Bcl6*, which is known to be subjected to SHM in germinal centre B cells and germinal centre derived B cell lymphoma [26], [27] and which was shown to accumulate AID dependent mutations also in mice [28]. Therefore, we retrovirally transfected primary AID deficient murine B cells with constructs encoding AID or K3 and sequenced a 600 bp fragment of the intronic *Bcl6* locus from GFP^+^ sorted cells. Strikingly, we found mutations in AID-transduced B cells (2 out of 44 independent sequences contained one mutation), whereas no mutations could be detected in K3 transduced B cells (p = 0.16, unpaired two-tailed t-test; Fig. 4D, S2). From these results we conclude that Lys22 of AID is more important for CSR than for IgV diversification but might also lead to an increase in off-target mutations.

## Discussion

AID mediates CSR and SHM by deaminating cytidine residues within the genomic DNA of the Ig locus. To keep off-target mutations low and to avoid genomic instability, AID has to be tightly targeted to the Ig locus and its activity regulated by several mechanisms, including restricted expression, nuclear/cytoplasmic shuttling, interaction with binding proteins and phosphorylation at Ser/Thr residues. Phosphorylation at Ser38 was shown to induce interaction with Replication Protein A, a DNA binding protein that is thought to mediate access of AID to transcribed DNA targets [29], [17]. Phosphorylation at Thr140 also regulates CSR and SHM as inhibition of phosphorylation at this residue decreases the biological activity of AID [18].

In this study, we aimed at assessing the role of lysine modification on AID activity. In previously published work it could be shown that nuclear AID is ubiquitinated at any of its eight lysine residues to initiate degradation. This was interpreted as means to keep nuclear AID levels low to avoid genomic instability [19]. However, conjugation of ubiquitin to cellular proteins not only initiates their destabilization but alternatively can modulate their specific function or their cellular trafficking [30], [31]. In this respect, we investigated the functional implication of single lysine to arginine mutants of the human AID protein and found that Lys22 is important in potentiating the specific activity of AID. While mutating Lys22 to Arg had no significant effect on AID activity in *E.coli* (Fig. 2) and on SHM in DT40 B cells (Fig. 4A, B, C), the implication on CSR activity was more pronounced, reflected in a significant reduction of IgG1 switched primary B cells (Fig. 1). Although Lys22 had no substantial effect on IgV diversification in DT40, off target *Bcl6* mutations were reduced in B cells expressing K3 compared to wt AID (Fig. 4D), implying that increased collateral damage might accompany a more efficient CSR activity.

As lysine-ubiquitination of AID was recently demonstrated to be involved in regulating AID stability/degradation [19], [32], we speculated whether a functional ubiquitin conjugation onto Lys22 could account for increased CSR activity of wildtype AID compared to mutant K3. In line with a recent publication, we found that Lys22 is a potential target of ubiquitin conjugation, however, ubiquitination is not restricted to a specific lysine residue as also mutant K3 was found to be ubiquitinated in transfected B cell lines (data not shown and [19]). In addition, lysine-independent N-terminal ubiquitination of AID as noticed especially upon impaired nuclear export [19] makes a clear-cut analysis of residue-specific ubiquitination difficult. Therefore, it is difficult to assess whether ubiquitination of Lys22 is actually responsible for the observed efficacy of wt AID compared to mutant K3 or whether other explanations account for the decreased effectiveness of mutant K3. In this regard it is conceivable that Lys22 is modified by other functional groups such as sumo, acetyl or methyl groups, or otherwise, that Lys22 has an intrinsic effect on CSR activity of AID. In either case, Lys22 could either affect targeting of AID to S-regions or the assembly of AID into a putative multiprotein-complex which executes CSR. In this regard it will be interesting not only to test for the presence of post-translational Lys22 modifications but also to analyze whether Lys22 mutation affects interaction with recently identified AID-interacting proteins such as CTNNBL1 [33], PKA [29], Spt5 [34], GANP [35], PTBP2 [36] and 14-3-3 adaptor proteins [37] which were found to recruit AID to its DNA target sites.

Notably, Lys22 is highly conserved as it can be found in AID from mammals and birds whereas AID from frog, bony- and pufferfish share an Arg residue at this position (Fig. S3). While SHM is present already in cartilaginous fish and is likely to occur in all jawed vertebrates, CSR appeared late in evolution and can be found only in land vertebrates, starting with amphibians [38]. Together with our observation that mutant K3 has more dramatic effects on CSR than SHM, this raises the possibility that Lys22 had co-evolved with the mechanism of CSR.

AID is regulated at several levels, including transcription, nuclear/cytoplasmic shuttling and phosphorylation. Our notion that Lys22 is important for proper AID function introduces lysine modification as a novel level of AID regulation. Future work will be required to unravel the role of Lys22 in mediating interactions with AID-binding proteins and to identify the presence of any functional lysine modifications at this position, such as sumoylation, methylation or acetylation.

## Materials and Methods

### Ethics statement

No ethics statement is required: the research described in this manuscript does not involve human participants. Also, no experimental procedures were performed on live animals. Mice used for this research were maintained at the animal facility of the University of Salzburg according to the national guidelines for animal welfare. Mice were killed by CO2 suffocation and spleens were used for further experiments.

### Class Switch assay

Class switching was analysed as previously described [20]. Briefly, B cells were purified from AID^−/−^ mice [3] and cultured in the presence of LPS and IL-4 for one day prior to infection with human AID (or variants) encoding retroviruses. CSR to IgG1 was analysed by flow cytometry three days after retroviral infection. For retrovirus production, Plat-E [39] cells were transfected with pMx-Ig-derived plasmids using Genejuice (Novagen). Lysine mutants of human AID were cloned into pMx-Ig plasmids using BamHI/XhoI sites and site-directed mutagenesis.

### Somatic Mutation

AID-induced IgV gene somatic mutation was monitored by measuring the frequency of sIgM-loss variants in AID^−/−^ ΨV^−/−^ sIgM^+^ DT40 cells [40] transfected with hAID-encoding vectors based on pExpressPuro2 (gift from J-M Buerstedde, Munich, Germany). Independent stable transfectants were expanded for 4 weeks and analysed by flow cytometry for the percentage of cells retaining surface IgM expression. For analysis of mutations in the IgV, genomic DNA was prepared from GFP+ sorted cells and DNA was amplified using specific primers (V-fwd: 5′-GGT GCA GGC AGC GCT GAC TCA GCC GGC CTC-3′; V-rev: 5′-CCA AAT CAC CAA AAA TCG ACA AAA TGT CAC-3′), and cloned using TOPO-cloning (invitrogen) followed by sequencing of independent clones.

Off-target mutations within the *Bcl6* intronic region were determined from primary retrovirally transfected mouse B cells. Briefly, primary resting B cells from AID deficient mice were retrovirally transfected with AID or K3 encoding constructs as described for CSR-assays. Transfected cells were sorted 3 days after transfection using FACS-sorting based on GFP expression and genomic DNA was isolated. PCR amplification of intronic *Bcl6* region was achieved using specific primers as described by Muto et al [28] (5′-CTT TCT TGG TTG GAG TCG AGG C-3′; 5′-CGG GCT TGA GGT CAT TTC TC-3′) followed by TOPO-cloning of purified PCR fragments and sequencing of several independent clones.

### AID-induced mutations in *E.coli*

Bacterial assays were performed essentially as described in Petersen-Mahrt et al. [4]. In brief, AID-induced mutations were monitored by measuring the frequency of Rifampicin (Rif) resistant clones that are generated by mutation of a Rif-sensitive allele in an Uracil-DNA Glycosylase (UNG)-deficient BW310 *E.coli* strain. Individual clones of BW310 cells transfected with empty vector or AID or K3 (AID^Lys22Arg^) expressing pTrc99 [4] based constructs were grown over night in 3 ml IPTG-containing medium at 30°C. Cultures were then spread on Rif and Ampicillin plates and the appearance of Rif-resistant clones was calculated (Mutation frequency).

### AID Localization

Constructs encoding AID or AID-variants fused C-terminally to GFP were generated by PCR-based mutagenesis and cloned into pEGFP-N1 (Clontech). The respective contructs were transiently transfected into HEK293T [41] cells in a six-well format using Genejuice and replated onto glass slides 24 h after transfection. Alternatively, stable transfectants of the mouse B cell line k46 [42], expressing pEGFP-N1 based AID-GFP fusion constructs were used. Nuclear export of AID was blocked by incubation of cells with Leptomycin B at a final concentration of 10 ng/ml for 3 h. Cells were fixed in 4% paraformaldehyde, permeabilized with 0.5% Triton X-100 and cell nuclei were stained using 1 µg/ml DAPI and covered in mounting medium (Vectashield).

### Western Blotting

Approximately 10e6 retrovirally transfected B cells or 10e5 DT40 cells were boiled in 50 µl of reducing SDS-sample buffer and subjected to SDS/PAGE. Samples were blotted onto PVDF membranes (Millipore) and detected by Western blot analysis using polyclonal rabbit serum specific for human AID (Abcam), GFP (Cell Signaling), or actin (Cell Signaling) and HRP-conjugated secondary antibodies specific for rabbit IgG (Dako).

## Supporting Information

Figure S1 **Mutations identified within the** ***IgV*** **gene from DT40 cells transfected with AID or K3, respectively.** The depicted sequence of the IgV gene was PCR amplified from transfected DT40 cells. Primer binding sites are underlined. Independent mutations are shown in uppercase letters above (AID; n = 21) and below (K3; n = 24) the IgV sequence, respectively. Mutations at hotspots (RGYW or WRCY motifs according to Rogozin and Kolchanov, 1992) are indicated as bold consensus sequence.
(DOC)

Figure S2 **Mutations identified within the** ***bcl6*** **gene from primary mouse B cells retrovirally transfected with AID or K3, respectively.** The depicted sequence of the *bcl6* intronic region was PCR amplified from retrovirally transfected and sorted mouse B cells. Independent mutations for AID (n = 44) are shown in uppercase letters above the bcl6 intronic sequence. No mutations were found for K3 (n = 49). (Accession No# NT_039624.7).
(DOC)

Figure S3 **Lys22 is conserved in higher vertebrates and birds.** Alignment of AID protein sequences from various species showing that Lys22 is conserved in birds and mammals. Species competent for switching are marked in bold. Sequences derived from M. Chatterji et al, J. Immunol. 2007;179;5274–5280.
(DOC)
