# Supplementary material for: Lysine Residue at Position 22 of the AID Protein Regulates Its Class Switch Activity
Source: PLoS One. 2012 Feb 20;7(2):e30667. doi: 10.1371/journal.pone.0030667 (PMC3282692; doi:10.1371/journal.pone.0030667)
Supplement: Figure S1 — Mutations identified within the IgV gene from DT40 cells transfected with AID or K3, respectively. The depicted sequence of the IgV gene was PCR amplified from transfected DT40 cells. Primer binding sites are underlined. Independent mutations are shown in uppercase letters above (AID; n = 21) and below (K3; n = 24) the IgV sequence, respectively. Mutations at hotspots (RGYW or WRCY motifs according to Rogozin and Kolchanov, 1992) are indicated as bold consensus sequence. (DOC) [file pone.0030667.s001.doc]

# Suppl Figure S1 IgVDT40 (revers)

## CCAAATCACCAAAAATCGACAAAATGTCACAATTTCACGA

## TGGGGGAAGAAAGACCGAgAcgAGGTCAGCGACTCACCTA

## GGACGGTCAGGGTTGTCCCgGCCCCAAATGCAgCACCACT

G

## GTTGTCTTCGT**AgCT**CCCACAgAAATAGACAGCCTCGTCA

## TCGGCTCGGACCCCAGTGATGGTTAATGTGGCTGTGGAGC

## CGGATTTGGAACCGGAgAATCGTGAAGGGATGTCCGAGGG

## TCTCTTGTCGTTGTCATAGATCACAGTGACAGGGGCACTG

## CCAGGAGACTTCTGCTGG**TACC**AGCCATAATAGTAACTTC

T

## A C

## C**AGCA**T**AGCT**GCCACCCCCGGAGCAGGTGATCTTGACGGT

CT

C

C

## TTCTCCTGGATTTGCTGACACCGAGGCCGGCTGAGTCAGC

## GCTGCCTGCACC
