# Supplementary material for: Lysine Residue at Position 22 of the AID Protein Regulates Its Class Switch Activity
Source: PLoS One. 2012 Feb 20;7(2):e30667. doi: 10.1371/journal.pone.0030667 (PMC3282692; doi:10.1371/journal.pone.0030667)
Supplement: Figure S2 — Mutations identified within the bcl6 gene from primary mouse B cells retrovirally transfected with AID or K3, respectively. The depicted sequence of the bcl6 intronic region was PCR amplified from retrovirally transfected and sorted mouse B cells. Independent mutations for AID (n = 44) are shown in uppercase letters above the bcl6 intronic sequence. No mutations were found for K3 (n = 49). (Accession No# NT_039624.7). (DOC) [file pone.0030667.s002.doc]

# Suppl Fig S2

# Bcl6 mouse, first intron (revers sequence)

1 TTCTCACTAC CCCTAGCACA AAAACTAAAT TTCTATGGGA GGGAGCGCAA CACAGAGATT

61 ATTTATTTTA CTGTTAAAAT AATATTGTTT GGTGCTGTTG TTTTTCATAG GGGACCTTTA

121 AAACTCATTT TTGCAACACT AGCTCCATCT CTCGCCAGGG TTCCAATAAC GCGGTATTAT

181 ACAGGCAACG CAACCCGCAG TTCTCAAGAC ATTTAACGTT CACTGTTTCC GGCAGGGGTG

241 GCCTCCAGCT CCCACTGCCC CCCAGGCAGC CCATTTCCTA GGTTCCAACT CTCAACCCAC

T

301 TCCCTCCCCG GGCCGCCGCC GCCGCTTTTC CCCATTCCTA CTCCCTCCCT TGAGGAGAGC

361 CACAGGTTGC AAATCCAACC AACCTCGCAA TCTGTTTTTG CAAAATCACT CACAAAGATC

C

421 TCCCTATCGC ACCCGCTCCT CCCGTCCGGG GTCCCCCAGC AGGAATCACA AAGTACCCCT

481 TCTTCCCTCC CTGATCTTGC AAATCAGGAA CGCAGGCTGG GGCTCTGTTT TTCTTCCTTC

541 TCCCAAAATA AGGACCTTGG GAATCTGAAG AGTGGCTATC GCTATACCCA GGGCCACTTT
