# Supplementary material for: Lysine Residue at Position 22 of the AID Protein Regulates Its Class Switch Activity
Source: PLoS One. 2012 Feb 20;7(2):e30667. doi: 10.1371/journal.pone.0030667 (PMC3282692; doi:10.1371/journal.pone.0030667)
Supplement: Figure S3 — Lys22 is conserved in higher vertebrates and birds. Alignment of AID protein sequences from various species showing that Lys22 is conserved in birds and mammals. Species competent for switching are marked in bold. Sequences derived from M. Chatterji et al, J. Immunol. 2007;179;5274–5280. (DOC) [file pone.0030667.s003.doc]

# 30

# Homo KFLYQFKNVRWAKGRRETYLC

Chimpanzee ------------K--------

Dog ----H-------K--H-----

Cow Q-----------K--H-----

# Mouse ----H-------K--H-----

# Chicken L---N---L---K--------

Frog ----HY--L---R--H-----

Zebrafish --IFHY------R--H-----

Catfish --I-HY------R--N-----

Pufferfish --I-HY------R--H-----
